# Improving Memory and Cognition by Enhancing Mitochondrial function and Biogenesis

**DOI:** 10.1002/alz.094581

**Published:** 2025-01-09

**Authors:** Dalia Nour, Mai Mostafa, Fatma Ismail, Rasha Hanafi, Reham M. AbdelKader

**Affiliations:** ^1^ German University in Cairo, Cairo Egypt; ^2^ German Univserity in Cairo, Cairo Egypt; ^3^ German univeristy in cairo, Cairo Egypt

## Abstract

**Background:**

Alzheimer’s disease (AD) treatment remains to be a challenge; this is due to the fact that the pathogenesis of the disease is very complicated and not well understood. Unquestionable is the fact that the pathological hallmarks of AD are accumulation of amyloid plaques, neurofibrillary tangles and mitochondrial dysfunction all leading to neuronal cell death. Mitochondrial dysfunction plays an important role in AD. Previous studies demonstrated reduced mitochondria number in hippocampal neurons in brains from AD patients, emphasizing the role of mitochondrial biogenesis in AD. They showed significant changes in proteins levels in AD brain tissues compared to controls, demonstrating reduced mitochondrial biogenesis leading to mitochondrial dysfunction. TIn this study our aim was test natural isolated compounds (Licochalcone(LicoA) and pyrroloquinoline quinone(PQQ)) with potential effect on mitochondrial biogenesis on memory and cognition in a neuroinflammatory mouse model.

**Method:**

A new GC/MS analysis method was developed and validated for detection of PQQ and LicoA in brain tissue. Mice were treated with either PQQ or LicoA in a neuroinflammatory mouse model. Memory was tested using object recognition and Y‐Maze tests. Brains were extracted from treated mice and levels of PQQ and LicoA were determined. Furthermore mRNA levels and proteins involved in mitochondrial biogenesis and function were determined using RT‐qPCR and ELISA.

**Result:**

For the first time we were able to develop a method for determination of PQQ and LicoA in brain tissue of mice. Interstingly both PQQ and LicoA were found to cross the blood brain barrier. Both PQQ and LicoA enhanced memory and cognition. Moreover, both PQQ an and LicoA increased the mRNA levels and the levels of proteins involved in the mitochondrial biogenesis pathway such as PGC‐1α, NRF‐1, ND1 and ATP levels.

**Conclusion:**

Isolated natural phenolic compounds such as PQQ and LicoA levels were determined in brain tissues of mice after peripheral adminstration. These compounds enhanced memory and cognitive function and thier mechanism of action involves enhancing mitochondrial biogenesis and function. Therefore, enhancing mitochondrial biogenesis may represent a new era for treatment of AD.

